# Machine learning-based analysis for prediction of surgical necrotizing enterocolitis in very low birth weight infants using perinatal factors: a nationwide cohort study

**DOI:** 10.1007/s00431-024-05505-7

**Published:** 2024-03-30

**Authors:** Seung Hyun Kim, Yoon Ju Oh, Joonhyuk Son, Donggoo Jung, Daehyun Kim, Soo Rack Ryu, Jae Yoon Na, Jae Kyoon Hwang, Tae Hyun Kim, Hyun-Kyung Park

**Affiliations:** 1 Department of Pediatrics, Hanyang University College of Medicine, 222 Wangsimni-ro, Seongdong-gu, Seoul, 04763 Republic of Korea; 2 Department of Pediatrics, Samsung Medical Center, Sungkyunkwan University School of Medicine, 81, Irwon-ro, Gangnam-gu, Seoul, 06351 Republic of Korea; 3 Department of Artificial Intelligence, Hanyang University, 222 Wangsimni-ro, Seongdong-gu, Seoul, 04763 Republic of Korea; 4 Department of Pediatric Surgery, Hanyang University College of Medicine, 222 Wangsimni-ro, Seongdong-gu, Seoul, 04763 Republic of Korea; 5 Biostatistical Consulting and Research Lab, Medical Research Collaborating Center, Hanyang University, 222 Wangsimni-ro, Seongdong-gu, Seoul, 04763 Republic of Korea; 6 Department of Computer Science, Hanyang University, 222 Wangsimni-ro, Seongdong-gu, Seoul, 04763 Republic of Korea

**Keywords:** Necrotizing enterocolitis, Very low birth weight, Machine learning, Neonatal intensive care unit

## Abstract

Early prediction of surgical necrotizing enterocolitis (sNEC) in preterm infants is important. However, owing to the complexity of the disease, identifying infants with NEC at a high risk for surgical intervention is difficult. We developed a machine learning (ML) algorithm to predict sNEC using perinatal factors obtained from the national cohort registry of very low birth weight (VLBW) infants. Data were collected from the medical records of 16,385 VLBW infants registered in the Korean Neonatal Network (KNN). Infants who underwent surgical intervention were identified with sNEC, and infants who received medical treatment, with medical NEC (mNEC). We used 38 variables, including maternal, prenatal, and postnatal factors that were obtained within 1 week of birth, for training. A total of 1085 patients had NEC (654 with sNEC and 431 with mNEC). VLBW infants showed a higher incidence of sNEC at a lower gestational age (GA) (*p* < 0.001). Our proposed ensemble model showed an area under the receiver operating characteristic curve of 0.721 for sNEC prediction.

*    Conclusion*: Proposed ensemble model may help predict which infants with NEC are likely to develop sNEC. Through early prediction and prompt intervention, prognosis of sNEC may be improved.

**Table:** 

**What is Known:** *• Machine learning (ML)-based techniques have been employed in NEC research for prediction, diagnosis, and prognosis, with promising outcomes.* *• While most studies have utilized abdominal radiographs and clinical manifestations of NEC as data sources, and have demonstrated their usefulness, they may prove weak in terms of early prediction.*	
**What is New:** *• We analyzed the perinatal factors of VLBW infants acquired within 7 days of birth and used ML-based analysis to identify which infants with NEC are vulnerable to clinical deterioration and at high risk for surgical intervention using nationwide cohort data.*

**Supplementary Information:**

The online version contains supplementary material available at 10.1007/s00431-024-05505-7.

## Introduction

Necrotizing enterocolitis (NEC) is a severe gastrointestinal disease with high morbidity and mortality rates in preterm infants. The incidence of NEC varies from 2 to 13% in very-low-birth-weight (VLBW) preterm infants [1]. Treatment of NEC typically starts with conservative treatment, such as bowel rest, gastric decompression, and broad-spectrum antibiotics. However, medical management is insufficient for patients with advanced NEC with a deteriorating clinical or biochemical status. In 27–52% of infants with NEC, surgical intervention, including primary peritoneal drains or laparotomy, is required [2–4]. The mortality rate of NEC ranges from 20 to 30%, with the highest rate occurring in infants requiring surgery [5–7]. The mortality rate of infants with surgically treated NEC (sNEC) is higher than that of infants with medically treated NEC (mNEC) and improves as gestational age (GA) increases [8–11]. Therefore, early detection and prompt treatment are critical to improve outcomes in infants with NEC.

Several predictive factors of sNEC have been reported in premature infants [12, 13]. Hassani et al. conducted a multicenter case-control study and reported low GA, early onset of NEC, low bicarbonate levels, and hemodynamically significant patent ductus arteriosus as independent risk factors for sNEC. Liu et al. reported similar results in a single-center study. However, despite these efforts, no consensus has been reached regarding the risk factors and prevention strategies for sNEC; consequently, the prognosis and mortality rates of sNEC have remained unchanged for decades.

Employing machine learning (ML)-based techniques has yielded promising results in many areas of NEC research, including prediction, diagnosis, and prognosis [14]. To date, few studies regarding the prediction of sNEC have been conducted [15–20]. Most of these studies used clinical data such as abdominal radiographs and clinical manifestations (bloody stools and abdominal distension) for training. The absolute indication for surgical intervention of NEC is bowel perforation, which can be diagnosed through abdominal radiography showing pneumoperitoneum. Thus, surgeons’ decisions are usually based on the radiographic signs as well as clinical manifestations in patients. However, even in the absence of signs of pneumoperitoneum, surgeons should consider surgical treatment when infants are in a severely deteriorating NEC state. Consequently, radiographic and clinical symptom data can be useful as decisive tools for sNEC; however, these may prove weak in terms of early prediction of sNEC. In this context, we aimed to analyze the perinatal factors of VLBW infants acquired within 7 days of birth and used ML-based analysis to identify which infants with NEC are vulnerable to clinical deterioration and at high risk for surgical intervention using nationwide infant cohort data.

## Materials and methods

### Participants

Data were collected from the Korean Neonatal Network (KNN) registry. The KNN is a nationwide registration system for VLBW infants in Korea that was officially established in April 2013. Seventy-seven hospitals with neonatal intensive care units (NICUs) in South Korea are part of the KNN. Using the data of VLBW infants in the KNN, the prevalence of morbidities and long-term outcomes in a large population of the country can be determined. Data from 16,385 VLBW infants born between January 2013 and December 2020 and registered in the KNN were analyzed in this study. Infants diagnosed with NEC grade ≥ II were included. Infant data without information on NEC were excluded from this study. Finally, the clinical data of 1085 VLBW infants were used for the incidence analysis of NEC.

For the risk factor analysis of sNEC in VLBW infants, the clinical information of patients in the KNN database was retrospectively analyzed. The inclusion process is illustrated in Online Resource 1. The infants who underwent surgical intervention (peritoneal drainage or laparotomy) for severe NEC were included in the sNEC group. In addition, infants with severe NEC requiring surgery but who were too unstable to undergo surgical treatment and died before surgery were placed in the sNEC group (*n* = 654). The infants who received medical treatment for NEC were included in the mNEC group (*n* = 431).

### Data collection and statistical analysis

A total of 38 perinatal factors were included in the ML algorithm. The incidence of sNEC according to GA was analyzed using the Cochran–Armitage trend test. Univariate analyses were performed to describe the characteristics of the study population and explore the association between these characteristics and sNEC. Continuous variables are presented as mean ± standard deviation (SD) and were compared using the *t*-test or Wilcoxon rank-sum test. Categorical variables are in the form of percentages and frequencies and were compared using the chi-square or Fisher’s exact test.

### Definitions

Each disease was defined according to the KNN manual of operations. NEC was defined according to the modified Bell’s staging classification grade ≥ II. Respiratory distress syndrome (RDS) was diagnosed based on both clinical and radiographic findings. Hypotension was diagnosed when medications were required to treat hypotension within 7 days of birth. Early onset sepsis was defined as a positive result on a blood culture performed before 3 days of birth, with clinical signs of infection.

### Data preprocessing for ML

The dataset used for the experiment contained 38 variable compositions. Data from a total of 1085 NEC patients, with 431 in the negative class and 654 in the positive class for sNEC, were included. Because most ML algorithms have difficulty in predicting when there are missing values, handling missing values is crucial for improving data quality and model performance. In this study, we added specific values to the missing values to handle missing values. Specifically, the variable “bbph” (hydrogen ion concentration in the blood within 1 h of birth) had the highest missing rate at 29.31%, and “bhead” (head circumference at birth), “bhei” (height at birth), and “btem” (body temperature at birth) had missing rates of 10.69%, 10.05%, and 6.54%, respectively. These four variables contained only continuous values, and their missing values were imputed using the means of the existing data. For the discrete variables “apgs1” (1-min Apgar score) and “apgs5” (5-min Apgar score), which had a missing rate of 1.11%, the missing values were imputed using the mode of the available data. Then, min-max normalization was applied to scale all variables between 0 and 1. Finally, to evaluate the ML models on limited data, *K*-fold cross-validation was used, and the dataset was divided into tenfold (i.e., *K* = 10). Therefore, the ratio between the training and validation sets was divided into 0.9 to 0.1. To ensure class balance in these sets, we used a stratified *K*-fold technique.

### Training

In this study, the sNEC prediction problem is cast as a binary classification problem, and thus, we used the binary cross-entropy (BCE) loss to train the proposed deep learning model. Determining appropriate hyperparameters during training is important. Specifically, the Adam optimizer [21] was used with a learning rate of 1e − 3 and a batch size of 128. In addition, as a regularization technique, dropout [22] was applied to the hidden layers at a rate of 0.2, and batch normalization [23] was used. To avoid overfitting, we used an early stopping technique, which stopped training if the minimum loss did not change for the last 10 epochs, instead of using a fixed number of iterations during training. The proposed deep learning models were developed using the PyTorch framework [24], and the evaluation metrics were implemented using the Scikit-learn library [25]. Specifically, different metrics such as the area under the receiver operating characteristic curve (AUROC), accuracy, precision, recall, and F1-score were used for performance evaluation. ROC curve analysis was performed to explore the trade-off between the true positive rate (TPR) and false positive rate (FPR) for comparison with other methods.

### Network configuration

The proposed model incorporates an ensemble technique by applying different initial seeds to the same architecture to improve the performance. The model selection process consisted of two stages. In stage 1, we explored different network configurations, including the number of hidden nodes, layers, and the adoption of parallel structures, and identified the optimal activation function. Our final model used preprocessed data as inputs for two separate branches. Each branch consisted of a single layer, and the layer had 38 input and 32 output nodes. The features from the two branches were then combined by summation to create a single hidden input. This single input was fed into a layer with 16 output nodes, and then the output served as the input to another layer with eight output nodes. The activation function used in all layers was PReLU. Finally, after passing through a linear layer, the output was subjected to a sigmoid activation function for binary classification. In stage 2, the ensemble approach was applied to the selected model to determine the number of models that give the best performance. We experimented with three, five, and seven models, each of which was initialized differently. Finally, seven models with different initializations yielded the best results. The final model is shown in Online Resource 2. We used the ensemble technique with soft voting, which contributed to performance improvement in our previous study [26] and observed improved classification results using it in a similar way. Therefore, we extended the ensemble technique in our study by changing the number of models to obtain an optimal score.

## Results

### Baseline characteristics of the patients

Among the 16,385 VLBW infants registered in the KNN database between 2013 and 2020, the incidence of NEC was 6.6% (1085/16,385). The baseline characteristics of the infants are presented in Online Resource 3. Among the 1085 infants diagnosed with NEC grade ≥ II, 654 infants had sNEC (60.3%) and 431 infants had mNEC (39.7%). Factors such as low GA, low birth weight (BW), intubation at initial resuscitation, RDS, and hypotension were significantly associated with sNEC.

### Trends in NEC incidence according to GA

**Fig. 1:**
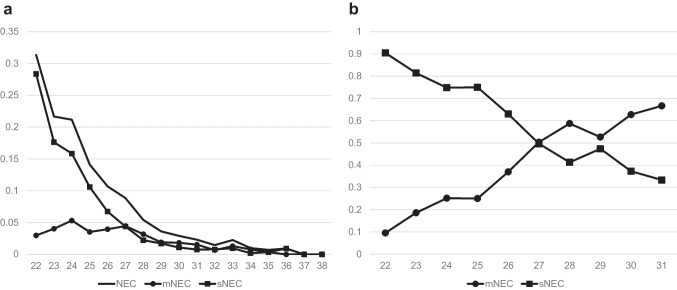
NEC incidence of VLBW infants according to GA (weeks). **a** Incidence of NEC, mNEC, and sNEC in VLBW infants according to GA. The rates of NEC, mNEC, and sNEC decreased as GA increased (NEC: *p* ≤ 0.001, mNEC: *p* ≤ 0.001, sNEC: *p* ≤ 0.001). **b** Proportion of mNEC and sNEC in VLBW infants between 22 and 31 weeks of GA. Prior to 27 weeks of GA, the ratio of sNEC was higher than that of mNEC, but after 27 weeks of GA, the ratio of mNEC became higher than that of sNEC. GA, gestational age; mNEC, medically treated NEC; sNEC, surgical NEC treated with surgery, or death before surgery with clinical evidence requiring surgery

The incidence of NEC was significantly associated with a lower GA (*p* < 0.001). The highest incidence of NEC (31.3%) was observed in infants with 22 weeks of GA, which decreased to 0.9% by 36 weeks (37–38 weeks, 0.0%). The incidence of sNEC decreased from 28.4% at 22 weeks of GA to 0.4% at 35 weeks of GA (36 weeks, 0.9%; 37–38 weeks, 0.0%). Trend analyses showed a significant decrease in the incidence of sNEC between 22 and 38 weeks of GA (Fig. 1a) (*p* < 0.001). In the extremely preterm infants (EPI) group (21–27 weeks of GA), the incidence of sNEC (9.3%) was higher than that of mNEC (4.2%) but not in infants with a GA > 27 weeks (1.3% vs. 1.8%, Fig. 1b).

### Performance of the proposed ensemble model

**Fig. 2:**
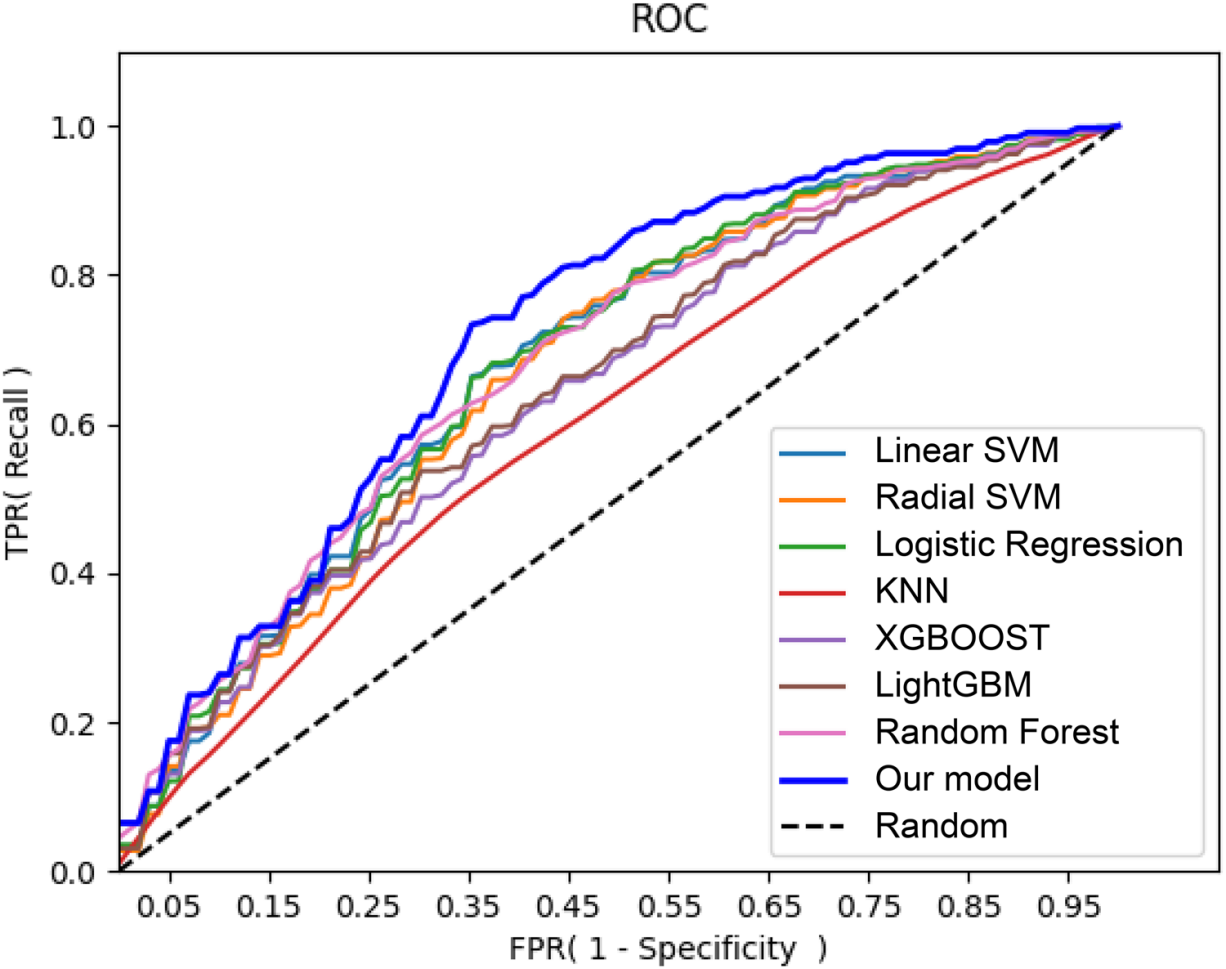
AUROC of sNEC prediction of ML models

The AUROC value of our model for predicting sNEC was 0.7210 (Fig. 2). The full performances of the proposed model and other conventional ML models are compared in Table 1. Our ensemble model outperformed all other conventional ML models in terms of the AUROC values, accuracy, and F1-score. Our ensemble model outperforms other conventional ML models in terms of sensitivity, positive likelihood ratio (PLR), negative likelihood ratio (NLR), positive predictive value (PPV), negative predictive value (NPV), F1-score, AUROC, global accuracy, and post-test probability. Random forest performs best in terms of specificity and PLR, as it is robust to noise with limited data.

**Table 1:** Performance metrics of the proposed model for prediction of sNEC

	**Sensitivity**	**Specificity**	**PLR**	**NLR**	**PPV**	**NPV**	**F1-score**	**AUROC**	**Global accuracy**	**Post-test probability**
Linear SVM	0.6812	0.6508	1.9911	0.4938	0.6905	0.6667	0.6771	0.6850	0.6812	0.5413
Radial SVM	0.6913	0.6430	1.9933	0.4864	0.6905	0.6698	0.6840	0.6736	0.6913	0.5410
Logistic regression	0.6857	0.6514	1.9943	0.4849	0.6894	0.6652	0.6822	0.6836	0.6857	0.5434
KNN	0.5012	0.6011	1.2938	0.8373	0.5963	0.5428	0.4544	0.6042	0.5012	0.4310
XGBOOST	0.6314	0.6353	1.7597	0.5833	0.6580	0.6186	0.6280	0.6461	0.6314	0.5118
LightGBM	0.6406	0.6489	1.8517	0.5557	0.6762	0.6364	0.6328	0.6534	0.6406	0.5246
Random forest	0.6656	0.6644	2.0607	0.5098	0.6925	0.6552	0.6597	0.6869	0.6656	0.5467
Proposed model	0.7049	0.6496	2.0297	0.4564	0.7010	0.6867	0.6983	0.7210	0.7049	0.5486

Pre-test probability is 0.6019

*PLR* positive likelihood ratio, *NLR* negative likelihood ratio, *PPV* positive predictive value, *NPV* negative predictive value, *SVM* support vector machine, *KNN* K-nearest neighbors, *XGBOOST* extreme gradient boosting, *LightGBM* light gradient-boosting machine

### Significant risk factors as analyzed using the SHAP method

**Fig. 3:**
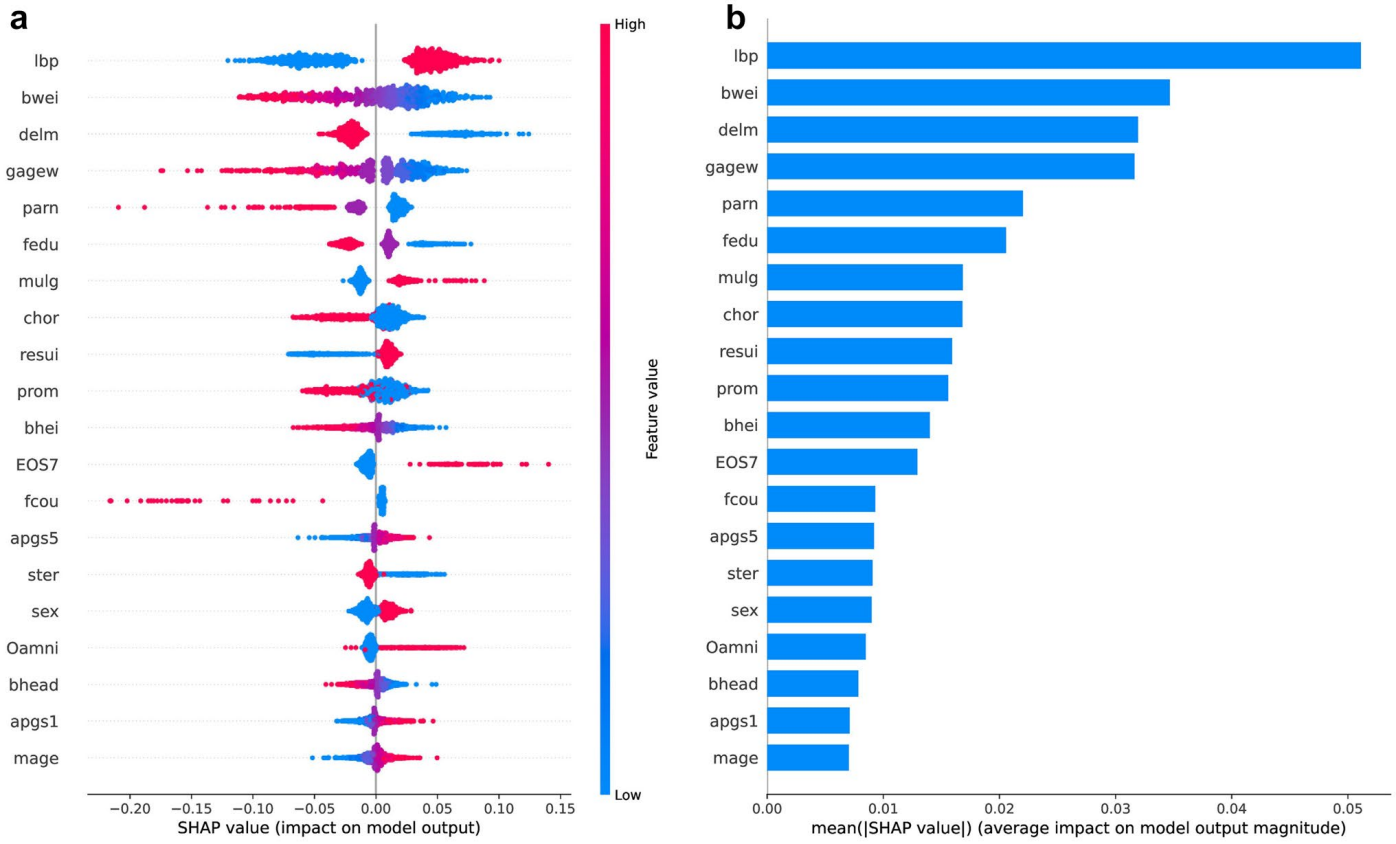
Top 20 variables contributing to sNEC prediction. **a** SHAP summary and **b** importance matrix plot. SHAP, Shapley additive explanations

A summary of the Shapley additive explanations (SHAP) and an important matrix plot for the prediction of sNEC are shown in Fig. 3. We identified the top 20 variables that contributed the most to the prediction of sNEC in VLBW infants. Hypotension, low BW, vaginal delivery, and low GA were the most influential variables for sNEC.

## Discussion

In this national cohort study, we developed an ML-based ensemble model to predict which VLBW infants with NEC are at risk for surgical intervention using perinatal variables obtained within 1 week of birth. Our analysis identified hypotension requiring medical treatment as the most significant variable associated with sNEC. In addition, we found that factors indicating the immaturity of patients, such as low GA and BW, had a significant impact on the prediction of sNEC.

The KNN data on VLBW infants in Korea demonstrated that the incidence of NEC was 6.6% and was inversely related to GA. Several investigators reported an increased incidence of NEC with decreasing GA and a higher incidence of NEC in EPIs [1, 5, 27]. We analyzed the incidence of the severe form of NEC requiring surgery or leading to death prior to surgery and confirmed that the incidence of sNEC was high in the EPI group (9.3%) and that low GA was the main risk factor for sNEC. This result regarding sNEC is similar to that reported previously [28]. Our analysis of NEC incidence (Online Resource 2) also demonstrated that the EPI group was more likely to undergo surgical rather than medical interventions for NEC. Several perinatal factors such as low GA, low BW, mode of delivery, antenatal steroids, and low Apgar scores have been identified as predictive factors for sNEC [8, 13, 29, 30]. Factors related to the early stages of the neonatal period, such as the use of a ventilator on the first day of life, the use of steroids and indomethacin in the first week of life, and breastfeeding, have also been associated with the incidence of sNEC [28]. Regarding the timing of NEC occurrence, 40% of NEC cases occur within 14 days of birth, and NEC cases that occur in the early postnatal period have a high rate of requiring surgical treatment than that for late-onset NEC [30–33]. In the present study, we confirmed that approximately 50% of all sNEC occurred within 14 days of birth. In conclusion, previous studies have shown that the development of NEC, especially with a high likelihood of sNEC, occurs early in life and that factors related to the perinatal period, such as GA, BW, and early postnatal treatment, are important risk factors for the development of sNEC. Considering that the generally known pathogenesis of NEC is related to intestinal immaturity and microbial colonization [5, 6], it can be assumed that perinatal factors have a significant influence as predictors of sNEC. This suggests that it may be possible to predict sNEC using perinatal and early postnatal factors.

To identify infants at high risk for sNEC by combining various factors that manifest during the early stages of the perinatal period, we developed a model utilizing various machine learning techniques to analyze a significantly large national cohort study dataset. The proposed ensemble method operates via a two-step process. In stage 1, we first identified the best deep learning model using various ML techniques on a limited number of datasets. In stage 2, the selected model was subjected to iterative soft voting with different weights to explore the optimal number of votes. As a result, our final model outperformed conventional ML models such as those by support vector machine (SVM) [34, 35], logistic regression [36], K-nearest neighbors (KNN) [37], extreme gradient boosting (XGBOOST) [38], light gradient-boosting machine (LightGBM) [39], and random forest [40] for the sNEC binary classification task, even with a limited dataset of factors available from VLBW infants.

However, the proposed ML model showed an AUROC value of 0.721, which was inferior to other results from ML-based studies regarding the prediction of sNEC. Given the significant influence of perinatal factors on the development of sNEC, this study focused on the possibility of identifying a high-risk group for sNEC during the early stages of birth. Other models with higher AUROC values include factors that reflect the infant’s condition at the time, such as clinical signs, blood test results, and radiological findings, as the basis for judgment [15–18]. In other words, these are useful models for diagnosing sNEC and NEC that have already progressed to a level that requires consideration of surgical treatment. They can be helpful in deciding whether to perform surgery for the treatment of NEC. However, extremely low BW or extremely preterm infants require minimal intervention. Therefore, the evaluation of blood tests or physical examinations is inevitably limited, and there may be situations in which it is difficult to apply prediction models using clinical signs, blood test results, and radiological findings to evaluate the disease state of infants in clinical practice. Early identification of infants at high risk of progression to sNEC is important. The mortality rate by sNEC is higher than that by mNEC [8–11], and surgical treatment of NEC is associated with significant growth delays and the risk of neurodevelopmental impairments. Compared to infants with mNEC, the risk is higher in infants with sNEC [41–43]. The early identification of infants who are likely to progress to sNEC helps in deciding policies and management, including decisions on the advancement of feeding and more aggressive treatment, or close observation when findings suggestive of NEC are discovered. Therefore, despite the relatively low AUROC of this model, it is thought to be useful for managing the treatment of VLBW infants in clinical practice. It predicts a high-risk group for the occurrence of sNEC or deterioration of NEC using factors identified in the early perinatal period.

This study has several limitations. The proposed ML model for predicting the high-risk group for sNEC was developed using only variables registered in the KNN database. Although the comprehensive coverage of all information on VLBW infants in Korea based on a large national cohort is a strength of this study, the proposed ML model can be improved by integrating more detailed early perinatal clinical information. Moreover, although SHAP allows us to identify variables with high risk factors, it does not provide insights into the dependencies or correlations between variables.

## Conclusion

NEC is one of the most serious gastrointestinal diseases with poor outcomes, especially in VLBW infants. The ensemble ML model described herein can assist clinicians in identifying infants at increased risk for sNEC, potentially leading to earlier diagnosis and prompt surgical intervention, leading to better prognosis and survival of patients in the future.

### Supplementary Information

Below is the link to the electronic supplementary material.

Supplementary file1 (PDF 233 KB)

## Data Availability

No datasets were generated or analysed during the current study.

## Abbreviations

AUROC: Area under the receiver operating characteristic curve
BCE: Binary cross-entropy
BW: Birth weight
EPI: Extremely preterm infants
GA: Gestational age
GDM: Gestational diabetes mellitus
IVF: In vitro fertilization
KNN: Korean Neonatal Network, K-nearest neighbor
LightGBM: Light gradient-boosting machine
ML: Machine learning
NEC: Necrotizing enterocolitis
NLR: Negative likelihood ratio
NPV: Negative predictive value
PIH: Pregnancy-induced hypertension
PLR: Positive likelihood ratio
PPV: Positive predictive value
PROM: Premature rupture of membranes
RDS: Respiratory distress syndrome
ROC: Receiver operating characteristic curve
SD: Standard deviations
SGA: Small for gestational age
SHAP: Shapley additive explanations
SVM: Support vector machine
XGBOOST: Extreme gradient boosting
