# Supplementary material for: Machine learning-based analysis for prediction of surgical necrotizing enterocolitis in very low birth weight infants using perinatal factors: a nationwide cohort study
Source: Eur J Pediatr. 2024 Mar 30;183(6):2743–51. doi: 10.1007/s00431-024-05505-7 (PMC11098869; doi:10.1007/s00431-024-05505-7)
Supplement: Supplementary file 1 — Supplementary file1 (PDF 233 KB) [file 431_2024_5505_MOESM1_ESM.pdf]

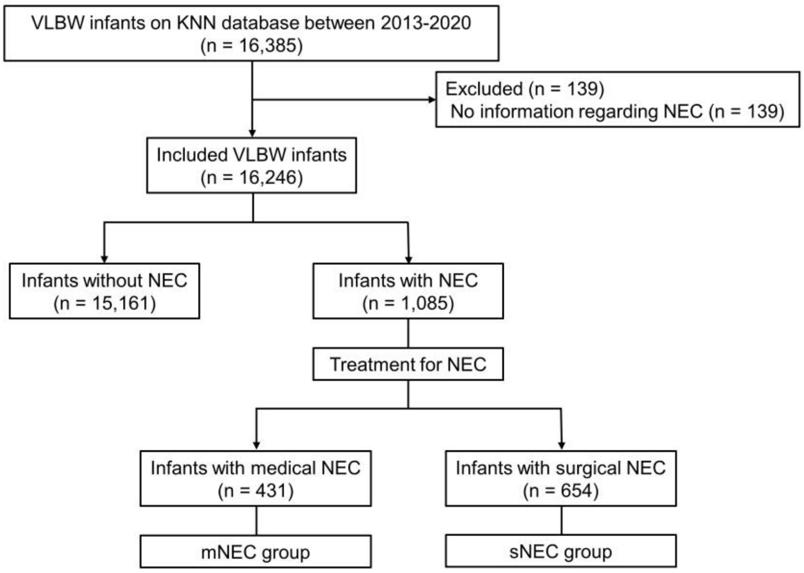


**Online Resource 1. Inclusion flow chart for machine learning-based analysis.** sNEC group, infants with severe NEC who were treated via surgical intervention including peritoneal drainage or laparotomy or those with clinical evidence of requiring surgery but died before surgery; mNEC group, infants who had NEC grade ≥II and were treated medically.

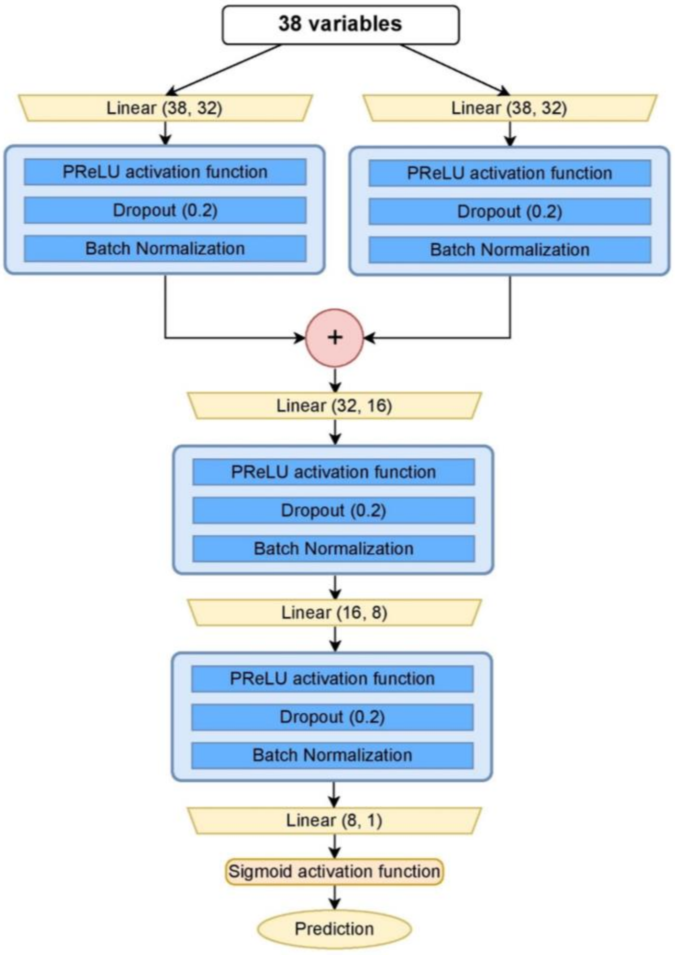


**Online Resource 2. Architecture of the ensemble model.**

**Online Resource 3. Baseline characteristics of infants included in the ML-based analysis**

| Variables | Total NEC (n = 1,085) | Medical NEC (n = 431) | Surgical NEC (n = 654) | p-value |
|---|---|---|---|---|
| **Neonatal factors** | | | | |
| Gestational age (weeks, mean ± SD) | 26.37 ± 2.55 | 27.23 ± 2.57 | 25.80 ± 2.37 | **<0.001** |
| Birth weight (gram, mean ± SD) | 876.95 ± 272.33 | 967.73 ± 283.82 | 817.13 ± 247.07 | **<0.001** |
| Male, n (%) | 595 (54.8) | 242 (56.1) | 353 (54.0) | 0.482 |
| SGA, n (%) | 180 (16.6) | 76 (17.6) | 104 (15.9) | 0.453 |
| Multiple birth, n (%) | 372 (34.3) | 136 (31.6) | 236 (36.1) | 0.124 |
| Cesarean section, n (%) | 824 (75.9) | 351 (81.4) | 473 (72.3) | **0.001** |
| Apgar score at 1 min (mean ± SD) | 3.86 ± 1.90 | 4.07 ± 1.90 | 3.71 ± 1.89 | **0.003** |
| Apgar score at 5 min (mean ± SD) | 6.19 ± 1.89 | 6.38 ± 1.85 | 6.07 ± 1.90 | **0.007** |
| Body temperature at birth (℃, mean ± SD) | 36.05 ± 0.64 | 36.08 ± 0.61 | 36.03 ± 0.66 | 0.059 |
| pH at birth (mean ± SD) | 7.24 ± 0.12 | 7.24 ± 0.11 | 7.24 ± 0.13 | 0.982 |
| **Maternal factors** | | | | |
| Maternal age (mean ± SD) | 33.33 ± 4.52 | 33.29 ± 4.65 | 33.35 ± 4.43 | 0.824 |
| Gravida (mean ± SD) | 1.98 ± 1.26 | 2.03 ± 1.35 | 1.94 ± 1.19 | 0.304 |
| IVF, n (%) | 255 (23.5) | 95 (22.0) | 160 (24.5) | 0.357 |
| GDM, n (%) | 73 (6.7) | 35 (8.1) | 38 (5.8) | 0.137 |
| PIH, n (%) | 176 (16.2) | 83 (19.3) | 93 (14.2) | 0.028 |
| Chorioamnionitis, n (%) | 352 (32.4) | 139 (32.3) | 213 (32.6) | 0.913 |
| PROM, n (%) | 422 (38.9) | 168 (39.0) | 254 (38.8) | 0.963 |
| Antenatal steroid, n (%) | 895 (82.6) | 364 (84.5) | 532 (81.3) | 0.186 |
| Oligohydramnios, n (%) | 145 (13.4) | 55 (12.8) | 90 (13.8) | 0.636 |
| **Clinical factors obtained within one week of birth** | | | | |
| Intubation at initial resuscitation, n (%) | 872 (90.4) | 313 (72.6) | 559 (85.5) | **<0.001** |
| Cardiac massage at birth, n (%) | 66 (6.1) | 24 (5.6) | 42 (6.4) | 0.565 |
| Epinephrine use at initial resuscitation, n (%) | 50 (4.6) | 19 (4.4) | 31 (4.7) | 0.799 |
| RDS, n (%) | 991 (91.3) | 376 (87.2) | 615 (94.0) | **<0.001** |
| Surfactant use, n (%) | 994 (91.6) | 379 (87.9) | 615 (94.0) | **<0.001** |
| Hypotension, n (%) | 577 (53.2) | 167 (38.7) | 410 (62.7) | **<0.001** |
| Inotropic use for hypotension, n (%) | 198 (18.2) | 56 (13.0) | 142 (21.7) | **<0.001** |
| Congenital infection, n (%) | 19 (1.8) | 7 (1.6) | 12 (1.8) | 0.796 |
| Early-onset sepsis, n (%) | 101 (9.3) | 27 (6.3) | 74 (11.3) | **0.005** |

**Abbreviations.** NEC, necrotizing enterocolitis; SGA, small for gestational age; pH, hydrogen ion concentration in the blood within 1 h of birth; IVF, in vitro fertilization; GDM, gestational diabetes mellitus; PIH, pregnancy-induced hypertension; PROM, premature rupture of membranes; RDS, respiratory distress syndrome
